# A Rare Cause of Left Ventricular Assist Device (LVAD) Obstruction: Left Atrial Dissection

**DOI:** 10.21470/1678-9741-2017-0060

**Published:** 2017

**Authors:** Michal Hulman, Panagiotis Artemiou, Alena Ftacnikova, Pavol Chnupa

**Affiliations:** 1 Medical Faculty of the Slovak Medical University, National Institute of Cardiovascular Diseases, Clinic of Cardiac Surgery, Bratislava, Slovakia.; 2 National Institute of Cardiovascular Diseases, Clinic of Cardiology, Bratislava, Slovakia.

**Keywords:** Heart Atria, Heart Aneurysm, Cardiac Surgical Procedures, Heart-Assist Devices

## Abstract

Left atrial dissection is a rare factor that may cause left ventricular assist device obstruction. Prompt diagnosis and surgical repair are essential. This case report describes our experience and a successful surgical management in a patient after HeartMate 3 implantation and mitral valve inflow obstruction due to a left atrial dissection.

**Table:** 

Abbreviations, acronyms & symbols	
ECMO	= Extracorporeal membrane oxygenation
LVAD	= Left ventricular assist device
RVAD	= Right ventricular assist device

## INTRODUCTION

Left atrial wall dissection is defined as the forced separation of layers of the left atrial wall by blood. It is a rare complication during cardiac surgery and it occurs in up to 0.84 percent of mitral valve replacements. This surgical complication can result in haemodynamic collapse and can also be fatal^[1,2]^.

Other rarer surgical complication causes include mitral valve repair, aortic valve surgery, myocardial infarction, percutaneous coronary intervention, cardiac mass excision, left ventricular aneurysm repair, coronary artery bypass graft, blunt cardiac trauma, pulmonary vein cannulation, infective endocarditis and spontaneous occurrence^[1,3]^.

Left atrial dissection can present a variety of symptoms such as chest pain, dyspnea, palpitations, fatigue, syncope, and cardiac arrest. One of the most common presentations is a rapid hemodynamic change with weaning of cardiopulmonary bypass. Obstruction of pulmonary veins and mitral inflow causes congestive heart failure and low-output syndrome^[4]^.

We are going to report a rare case of left atrial dissection related to a left ventricular assist device (LVAD) implantation, that caused obstruction of the assist device and right ventricular dysfunction, and discuss its management.

## CASE REPORT

A 38-year-old male patient was presented with end-stage heart failure, based on the New York Heart Association class IV, and was hemodynamically supported by a peripheral femoral venoarterial extracorporeal membrane oxygenation (ECMO) Cardiohelp device (Maquet Holding, Germany). The patient's medical history indicated that he has suffered from chronic heart failure, due to dilated cardiomyopathy, and that during the last two months, he has been hospitalized several times due to acute heart decompensation. ECMO was implanted as a bridge-tobridge strategy and he was transferred to our institution for further treatment. Accordingly, the patient underwent implantation of a LVAD, HeartMate 3 (Thoratec Corp, Pleasanton, CA, USA). The operation was performed through median sternotomy with ECMO support. The inflow cannula of the HeartMate 3 LVAD was implanted in the left ventricular apex, and the outflow cannula in the ascending aorta. After completion of the operation, and during weaning of the ECMO device based on signs of right ventricular dysfunction, a right ventricular assist device (RVAD), Cardiohelp (Maquet Holding, Germany), was implanted with cannulation of the femoral vein and the pulmonary artery.

During the immediate postoperative period, there were signs of LVAD low-flow due to low left ventricular filling and an increasing need for RVAD support. Additionally, serial transthoracic echocardiograms showed a hyperechogenic lesion in the left atrium that resulted in mitral valve inflow obstruction.

**Fig. 1:**
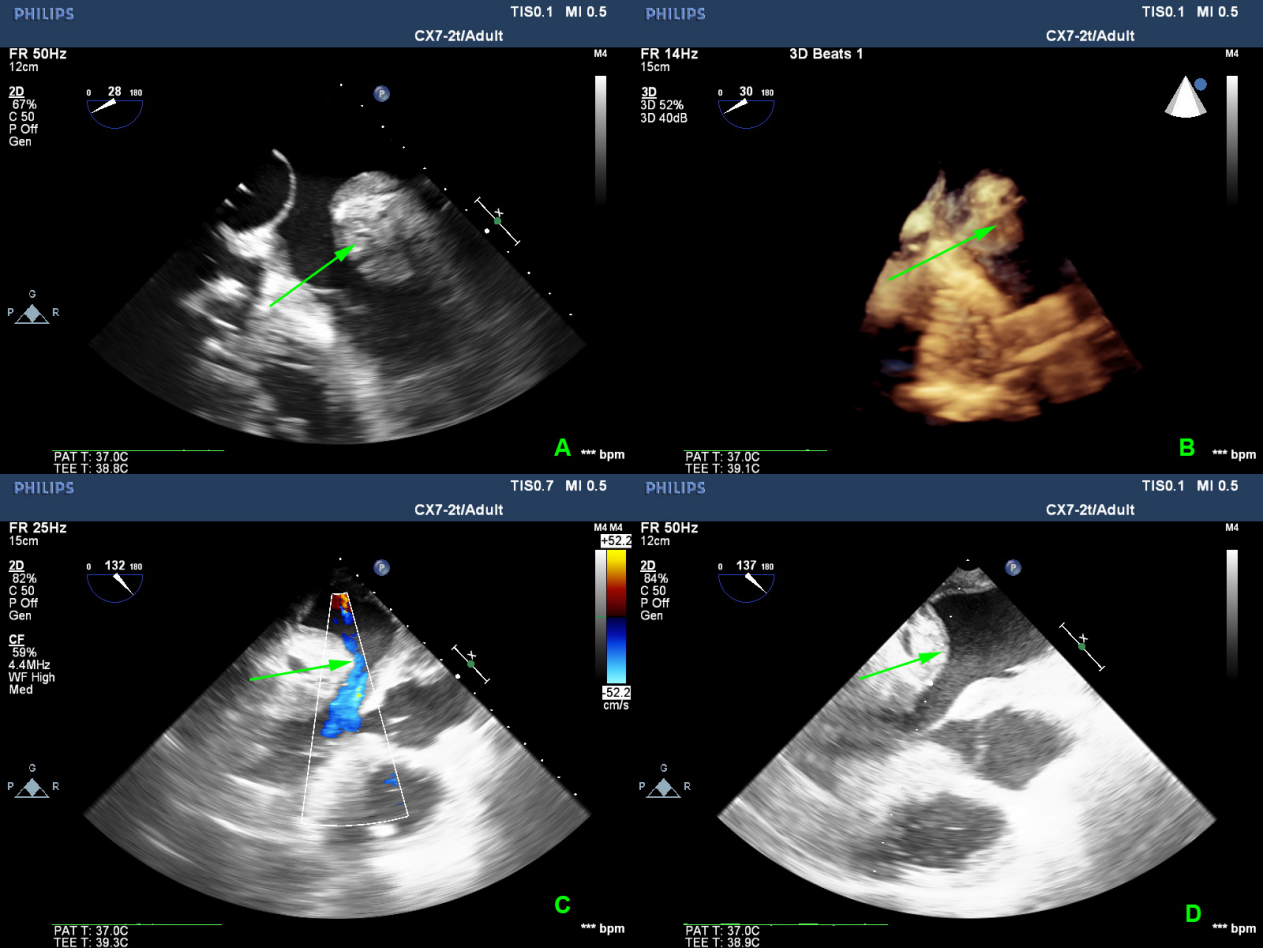
(A) Intraoperative echocardiogram showing left atrial dissection; (B) 3D echocardiography; (C) Intraoperative Doppler echocardiography; (D) Early postoperative echocardiogram after the repair.

Surgical revision was decided and intraoperative echocardiography confirmed the presence of left atrial lesion with the endocardial layer of the left atrium separated from the left atrial wall (Figures 1A, B and C). With the use of cardiopulmonary bypass, a left atriotomy was performed, which identified that the posterior atrial wall has suffered a large hematoma (atrial wall dissection) that obstructed the mitral valve inflow. The space was evacuated and the dissected layers were reapproximated using full thickness 4/0 polypropylene sutures to the posterior muscular wall through the mitral annulus and the left atrial appendage. Early postoperative transesophageal echocardiography showed that the left atrial lesion was significantly smaller, without any obstruction of the mitral valve inflow (Figure 1D). Also, during the procedure, the inflow cannula of the RVAD was transferred from the femoral vein to the right atrium.

After the left atrial wall dissection repair, the LVAD flow returned to normal and a weaning process for the RVAD was initiated. In the subsequent postoperative period, there was a recovery of the right ventricular function, and eventually the RVAD was explanted.

**Fig. 2:**
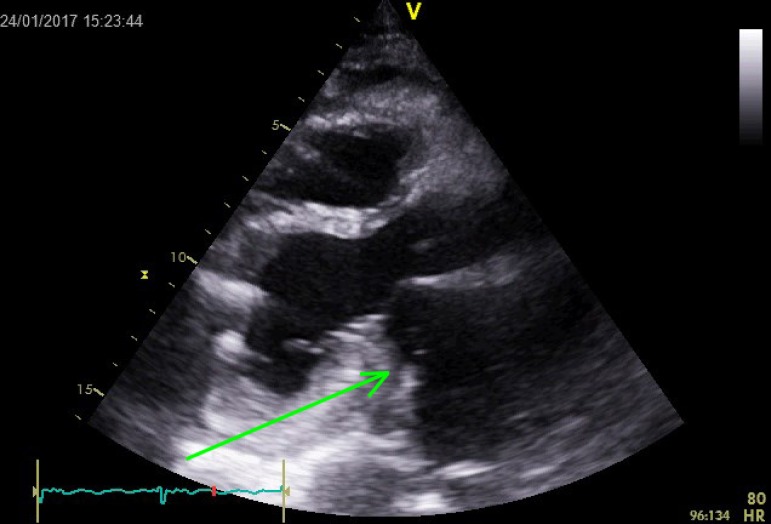
Follow-up echocardiogram showing residual thickening of the left atrial wall.

Moreover, the postoperative recovery was complicated, with few surgical revisions due to pericardial tamponade. On postoperative day 43, the patient was discharged in stable clinical condition. Transthoracic echocardiography on discharge showed regression of the atrial hematoma after the repair with residual thickening of the left atrial wall, without any obstruction of the mitral inflow (Figure 2). An informed consent and an institutional review board permission were obtained to present this case.

## DISCUSSION

Left atrial wall dissection is a rare complication and the true incidence, etiology and management remain poorly defined. It is mostly associated with atrioventricular junction injuries, however, as presented in this report, there are other less common cases remote from the atrioventricular junction (dome of left atrium or junction of the pulmonary veins entering the left atrium)^[4]^. We believe that the left atrial dissection in the presented case occurred during elevation and placement of the laparotomy pads under the heart while inserting the inflow cannula of the HeartMate 3 device into the left ventricular apex. Manipulation of the heart and mechanical pressure applied on the left atrium by the laparotomy pads may have resulted in left atrial wall dissection.

In 1985, Maeda et al.^[5]^ firstly reported a surgical repair of left atrial dissection through left thoracotomy. Since then, different surgical approaches have been described^[4]^. Surgical techniques reported include primary repair, repair with bovine/synthetic patch and in association with mitral valve replacement, prosthesis explantation and reimplantation for which careful sizing of the valve is crucial^[4,6,7]^.

Repair of the left atrial wall dissection should focus on evacuation of the hematoma and blood from the false atrial cavity, obliteration of the dissected cavity, and repair of the dissection entry point as required. In this case, we did not identify the dissection entry point, and, as such, a full-thickness continuous suture was used to obliterate the cavity. Furthermore, BioGlue (Cryolife, INC, Kennesaw, GA, USA) can be used to bring the dissected layer together^[1]^.

A spectrum of clinical presentations exists as well as required treatments that depend on how extensive the atrioventricular disruption is and on what is the direction of the dissecting blood. Prompt diagnosis and repair with obliteration of the false cavity may prevent obstruction of the mitral valve inflow, pulmonary artery hypertension and right heart failure. Based on the review of the available literature, all of intraoperative left atrial wall dissections should be repaired. Acute, left atrial wall dissections resulting in mitral valve inflow obstruction require repair; and delayed left atrial wall dissection in a stable patient can be managed conservatively^[1,4,8]^.

In conclusion, this study report describes a rare case of a left atrial wall dissection in association with an LVAD implantation, which was successfully managed with surgical repair.

**Table:** 

Authors' roles & responsibilities	
MH	Agreement to be accountable for all aspects of the work in ensuring that questions related to the accuracy or integrity of any part of the work are appropriately investigated and resolved; final approval of the version to be published.
PA	Drafting the work or revising it critically for important intellectual content; substantial contributions to the conception or design of the work; or the acquisition, analysis, or interpretation of data for the work; final approval of the version to be published.
AF	Substantial contributions to the conception or design of the work; or the acquisition, analysis, or interpretation of data for the work; final approval of the version to be published.
PC	Substantial contributions to the conception or design of the work; or the acquisition, analysis, or interpretation of data for the work; final approval of the version to be published.
